# Exploring the promoting effect of working time reduction on life satisfaction using Germany as a case study

**DOI:** 10.1057/s41599-022-01480-2

**Published:** 2022-12-17

**Authors:** Qinglong Shao

**Affiliations:** Institute of Chinese Studies, Freie Universität Berlin, Fabeckstr. 23-25, 14195 Berlin, Germany

**Keywords:** Social policy, Economics

## Abstract

Worktime reduction’s effect on life satisfaction is an important issue but one that has not been fully studied. This article fills this gap and uses an ordered probit model to analyse the working time reduction impact on life satisfaction in Germany by using the European Social Survey data, the mediating effect of health and cross-partner effect are also explored. A significantly negative correlation between working time and life satisfaction are revealed, showing that a short working week can improve Germans’ life satisfaction. Health is confirmed to be the important intermediate variable in the ‘worktime–health–life satisfaction’ nexus and about 28% of the satisfaction among German people is due to the change in health explained by working hours. Further, we find that high-earners prefer to work long hours whereas low-earners tend to work less; middle-earners show no personal preferences. Cross-partner effects are confirmed, as a male’s short working week can satisfy their partner, while a female’s long working hours can improve their partner’s life satisfaction. In light of this, working hours should be restricted to avoid unsatisfaction induced by overtime work and overtime compensation regulations should be strictly implemented, policy-makers also need to take gender differences into consideration.

## Introduction

Except for the conventional views that income plays an essential role in enhancing life satisfaction (Knabe and Rätzel, 2010; Sekulova and van den Bergh, 2013), worktime has become a key concept for satisfaction enhancement in recent years with the increasing focus on individual rights and work–life balance (Noda, 2020; Bolli and Pusterla, 2022). The worktime reduction impact on the satisfaction of life has been confirmed by multiple studies (Knight et al., 2013; Pullinger, 2014). Okulicz-Kozaryn (2011) is, as far as we know, the first to empirically examine such a relationship. The result shows that Europeans are tending to work less compared to Americans, and the author explained that Americans care more about the work outcome whereas Europeans place more value on the process. In a later study, Valente and Berry (2016) further reveal that Latin Americans prefer part-time jobs while US citizens prefer working longer hours. This is compatible with the finding of Okulicz-Kozaryn (2011) that US employees work harder. In the reality, the correlation between working time and life satisfaction may be inverted U-shaped (Collewet and Loog 2015), that increasing working hours can enhance well-being first but decline beyond the peaking point. In addition, women prefer part-time jobs to enable them to take care of the children or to develop their own interests (Booth and Van Ours, 2008, 2009; Rudolf, 2014), whereas men tend to work full-time or even overtime to feed their families or to pursue success (Okulicz-Kozaryn, 2011; Holly and Mohnen, 2012). Considering these gender differences in working hours, the cross-partner effect exists to gain a work–life balance (Booth and Van Ours, 2009).

To our knowledge, although researchers have explored the cases of Germany due to its large population size and economic scale in Europe (Pouwels et al., 2008; Knabe and Rätzel, 2010; Burgoon and Raess, 2011), they have simply discussed the role of hours of work in the income–satisfaction nexus, only a few studies have comprehensively examined the nexus between life satisfaction and the working hours of Germans people (including partnered couples). In addition, as a potentially important mediating variable in the worktime impact on the life satisfaction process, health is seldomly discussed and no previous studies estimated how much it accounts for (Bell et al., 2012; Wu, 2016; Muthuri et al., 2020). Therefore, this article aims to explore the effect of working hours on life satisfaction in a German sample. In light of this, the study makes four contributions: first, this study confirms the promoting effect of reduced working hours on life satisfaction in Germany; second, as far as we know, we are the first to estimate how much health is explained in the ‘worktime–health–life satisfaction’ nexus for Germans; third, income levels influence preferences on working hours in Germany, with high-earners tending to work more and low-earners preferring more leisure hours; fourth, cross-partner effects are detected, with both wives’ and husbands’ hours of work strongly impacting on their partners.

This paper is organised as follows. The section “Worktime effect on life satisfaction and the cross-partner effect: a brief overview” presents an overview of the worktime-satisfaction literatures. The section “Data and method” describes the data and methods employed in this study. Empirical results and robustness checks are shown in the section “Empirical results and analyses”, and the section “Discussion” makes the discussions. The last section concludes and proposes policy suggestions.

## Worktime effect on life satisfaction and the cross-partner effect: a brief overview

Overtime work is one of the hotly debated topics in worktime-satisfaction research, and previous empirical studies revealed that overtime work could significantly reduce workers’ satisfaction with life (Holly and Mohnen, 2012). In general, different types of overtime work led to different feelings of satisfaction. The satisfactory effect of mandatory overtime work depends on the interplay of the positive effects of extra income and a sense of achievement as well as the negative effects of work–life imbalance and work stress (Golden and Wiens-tuers, 2006). In most cases, the mandatory long working hours dissatisfied the workers. It is another story for voluntary overtime work. A study in Australia shows that employed fathers with children usually prefer longer working hours because they could earn overtime pay and achieve the feeling of accomplishment, which all contribute to increased well-being (Weston et al., 2004).

Moreover, the worktime effect on satisfaction differs in various countries and worktime types. Due to the different labour market structures, French employees are usually satisfied with longer working hours, while British employees prefer a shorter working week (Clark and Senik, 2006). The welfare system and tax revenue system play the roles, as better welfare care and a higher tax rate tend to promote employees to choose a shorter working week. Part-time jobs can sustain a work–life balance, particularly for women. Evidence shows that household wives with children are happier if they can do a job of <15 h per week (Booth and Van Ours, 2008). Couples without children find that part-time jobs make men happier, while worktime has no impact on women’s life satisfaction. In general, leisure hours are more important for men than women in enhancing life satisfaction (Noda, 2020).

The cross-partner effects of working hours on life satisfaction is another issue attracting considerable attention. Booth and Van Ours (2009) point out that husbands having full-time jobs can enhance wives’ happiness. It is plausible that flexible working schedules may satisfy women’s non-working needs for taking care of the children, while their spouses’ full-time jobs guarantee sufficient resources. Meanwhile, men anticipate better future prospects from full-time jobs. Likewise, Korean husbands’ short worktime may decrease their wives’ life satisfaction, but there appears to be no significant influence of Korean wives’ worktime on their husbands’ life satisfaction (Rudolf, 2014). This can be explained by the cultural norm in Korea that the husband plays a dominant role and shoulders the main responsibility within a family. A similar situation appears by using the American Time Use Survey in 2012–2013, empirical results show that increased time spent on work with American and Britons spouses could boost their life satisfaction, but increased time spent alone decreases single people’s life satisfaction (Hamermesh, 2020). In general, people expect their spouses or partners to work longer hours to support the family. Local culture and income levels play a key role.

## Data and method

### Data and descriptive statistics

Germany was selected as this study’s focal country because it has the largest population size in the EU (about 82.69 million in 2017: World Bank, 2022), and thus contributes a comparatively comprehensive sample to the dataset of the European Social Survey (ESS, 2017). Further, Germany has a good record in reducing working hours, with the average annual working time per worker declining from 1973 h in 1970 to 1376 h in 2016, which is the lowest in Europe (TCB, 2019).

The study’s data are generally sourced from the ESS, which aims to collect the public attitudes and values within Europe. It has developed a series of European social indicators, including attitudinal indicators (ESS, 2017). We use the eighth-round survey performed during 2016–17. The life satisfaction question reads: *All things considered, how satisfied are you with your life as a whole nowadays?* We use this as the dependent variable in our analysis. The independent variables are paid and unpaid working hours per week. Individuals who refuse to answer (‘Refusal’), leave answers blank (‘No answer’), or answer that they do not know (‘Don’t know’) are dropped. We finally generate 2852 valid respondents in Germany. Detailed survey questions and descriptions of the indicators are listed in Supplementary Information Table S1. Besides the life satisfaction and worktime variables, we also include personal characteristic indicators, such as gender, age, education, social inclusion, income, and health condition. In addition, we present life satisfaction distribution in terms of marital status, sex, income and working time categories in Table S2. Descriptive statistics and correlation matrix are presented in Tables S3 and S4, please see the Supplementary Information.

### Model specification

The outcome variable of this study, i.e., life satisfaction for Germans, is ordinal. This is intended to facilitate marginal effect analysis and discussion; the cross-sectional data structure also enables the ordered probit model as a suitable research method. More importantly, the ordered probit model takes the unobserved heterogeneity and ordinarily life satisfaction scales into consideration while using full information of the data (Rudolf, 2014). Since both the ordered probit and ordered logit models are commonly employed to analyse such ordinal data, we choose the former since it is widely used in related literature (Alesina et al., 2004; Clark and Senik, 2006; Bosselmann, 2012; Litchfield et al., 2012; Maitra and Rao, 2015; Yen and Zampelli, 2017; Kumar and Shetty, 2018; Alemi et al., 2019), and the ordered logit model is used to test robustness. The basic equation of the ordered probit model is

1 $$y_i^ \ast = \beta _iX_i + \varepsilon _i$$

where *y*_*i*_ stands for the dependent variables while $y_i^ \ast$ are the latent variables. According to Everitt (1984), latent variables are variables that are not directly observed but are rather inferred through a mathematical model from other variables that are observed. *X*_*i*_ is a vector of explanatory variables assessing the attribution of life satisfaction, and *β*_*i*_ is the coefficient of *X*_*i*_, a vector of estimated parameters to be projected, representing the impact magnitude of the independent on the dependent. Finally, *ε*_*i*_ is an unobserved white-noise disturbance, with *E*(*ε*_*i*_) = 0.

As this study has five-level of life satisfaction, we assume that *α*_1_, *α*_2_, *α*_3_, and *α*_4_ are thresholds to be projected, and *α*_1_ < *α*_2_ < *α*_3_ < *α*_4_, then based on Eq. (1) we generate the following Eq. (2):

2 $$y_i = \left\{ {\begin{array}{*{20}{c}} 1 & {y_i^ \ast \le \alpha _1} \\ 2 & {\alpha _1 < y_i^ \ast \le \alpha _2} \\ 3 & {\alpha _2 < y_i^ \ast \le \alpha _3} \\ 4 & {\alpha _3 < y_i^ \ast \le \alpha _4} \\ 5 & {y_i^ \ast > \alpha _4} \end{array}} \right.$$

The parameters of the model specified in Eq. (2) are estimated using the maximum-likelihood method. However, the coefficients of the models cannot reveal the effects of the regressors, so a marginal effect analysis is necessary to examine the effects of independent variables on the probability of each of the five different levels of life satisfaction. A partial change in the predicted probability of the outcome variable for a change in an explanatory variable at the mean value is specified as follows:

3 $$\left\{ {\begin{array}{*{20}{c}} {\partial p_1/\partial x_k = - \beta _k{\Phi}\left( {\alpha _2 - X_i\beta _i} \right)} \\ {\partial p_j/\partial x_k = \beta _k{\Phi}\left( {\alpha _{j - 1} - X_i\beta _i} \right) - {\Phi}\left( {\alpha _j - X_i\beta _i} \right)} \\ {\partial p_5/\partial x_k = \beta _k{\Phi}\left( {\alpha _5 - X_i\beta _i} \right)} \end{array}} \right.$$

We construct the following alternative econometric specification in order to estimate how much health can be explained in the worktime–satisfaction nexus:

4 $${\rm {Satisfaction}}_i = \alpha {\rm {Worktime}}_i + {\sum} {{\rm {Individual}}_i} + \mu _i$$

where Satisfaction_*i*_ is the life satisfaction level reported by individual *i*, Worktime_*i*_ is the reported working hours per week, ΣIndividual_*i*_ is the vector of respondents’ individual characteristics, including income, health, gender, age, education, and social inclusion, and *μ*_*i*_ is an error term.

## Empirical results and analyses

### Empirical analysis of worktime impact on life satisfaction and the marginal effects

Table 1 presents the empirical results of the relationship between worktime and satisfaction in life. In Model 1, the weekly worktime significantly correlates with the dependent variable, showing that fewer working hours can improve Germans’ general satisfaction with life. On the one side, Germans have a cultural norm of familyism and are happier working fewer hours to have more time to discharge family responsibilities and enjoy family relationships (Valente and Berry, 2016). On the other side, the income tax rate is high in Germany. In fact, it is already empirically verified that Europeans generally favour a shorter working week compared to workers in other advanced economies, such as the US (Alesina et al., 2004, 2006; Prescott, 2004; Okulicz-Kozaryn, 2011). This is consistent with the downward trend of annual working time per worker over the past half-century in EU-15 countries.

**Table 1:** Effect of worktime per week on self-reported life satisfaction using ordered probit model and the marginal effects.

Variables	Model 1: Ordered probit	Model 2: OLS	Model 3: OLS	Model 4: Marginal effects				
				Very unsatisfied	Unsatisfied	Fairly satisfied	Satisfied	Very satisfied
Worktime	−0.0023* (0.002)	−0.0018** (0.001)	−0.0013* (0.001)	0.0001* (0.000)	0.0001* (0.000)	0.0007* (0.001)	−0.0003* (0.000)	−0.0005* (0.000)
*Individual characteristics*								
Gender	−0.0620 (0.046)	−0.0265 (0.030)	−0.0378 (0.029)	0.0011 (0.001)	0.0023 (0.002)	0.0184 (0.014)	−0.0092 (0.007)	−0.0126 (0.009)
Age	0.0072*** (0.001)	0.0018** (0.001)	0.0044*** (0.001)	−0.0001*** (0.000)	−0.0003*** (0.000)	−0.0021*** (0.000)	0.0011*** (0.000)	0.0015*** (0.000)
Education	−0.0092 (0.030)	0.0087 (0.019)	−0.0050 (0.019)	0.0002 (0.001)	0.0003 (0.001)	0.0027 (0.009)	−0.0014 (0.004)	−0.0019 (0.006)
Social inclusion	0.1364*** (0.025)	0.1188*** (0.016)	0.0879*** (0.016)	−0.0024*** (0.001)	−0.0051*** (0.001)	−0.0404*** (0.007)	0.0202*** (0.004)	0.0277*** (0.005)
Income	0.0684*** (0.009)	0.0539*** (0.005)	0.0430 (0.005)	−0.0012*** (0.000)	−0.0025*** (0.001)	−0.0203*** (0.003)	0.0102*** (0.001)	0.0139*** (0.002)
Health	0.3448*** (0.027)		0.2181*** (0.017)	−0.0060*** (0.001)	−0.0128*** (0.002)	−0.1023*** (0.008)	0.0512*** (0.004)	0.0699*** (0.006)
Cons		3.0575*** (0.072)	2.2880*** (0.091)					
No. of obs.	2488	2489	2488					
Pseudo-*R*^2^	0.0626	0.0701	0.1306					

Standard errors in parentheses; *, **, and *** denote significant *p*-values at 10%, 5%, and 1% levels, respectively.

As for the control variables, income is one of the important promoting forces of life satisfaction. This effect has been confirmed by numerous previous studies (Diener et al., 1993; Diener and Biswas-Diener, 2002; Di Tella et al., 2003; Pouwels et al., 2008; Pedersen and Schmidt, 2011), and is generally interpreted as ‘more income brings greater happiness’ (Easterlin, 2001, p. 465). Consistent with prior studies, age is found to positively impact life satisfaction, implying that elders are generally happier than the young (Pouwels et al., 2008; Valente and Berry, 2016). This is influenced by the excellent social welfare system in Germany, as well as the wealth accumulated over time. Life satisfaction is also strongly affected by the frequency of engaging in social activities (Becchetti et al., 2012); ‘the greater the extent of participation, the greater the degree of happiness reported’ (Phillips, 1967, p. 479). In fact, work is also a kind of social participation. Isolation from society, for example, induced by the COVID-19 pandemic, may cause a negative impact on life satisfaction (Clair et al., 2021). Health is also found to be an important factor in enhancing life satisfaction (Booth and Van Ours, 2008; Pouwels et al., 2008); our estimation result reveals a significant relationship at the 1% level. For Americans, it is declining health that primarily drives down life satisfaction beyond midlife (Easterlin, 2006).

Gender and education indicators show no significant correlations with the dependent variable, which differs from the findings of Di Tella et al., (2003), reflecting that gender equality and education situations vary across countries. Germany ranked 3rd on gender equality in the 2014 Gender Inequality Index (EIGE, 2022). This may explain why no significant gender difference in life satisfaction is observed. Besides, people with a higher education level are more likely to have a higher salary and social status, but also have more responsibilities and heavy burdens, potentially producing no net effect on life satisfaction.

According to Wu (2016), health is an important intermediate variable in the worktime–satisfaction nexus. Accordingly, we try to measure to what extent weekly working hours may change life satisfaction via the health condition, thus testing the worktime–health–life satisfaction nexus. The coefficients of ordered probit regressions cannot directly reveal the regressors’ effects on each of the five different levels of life satisfaction (Islam et al., 2017), while the regression results of the ordered probit are very similar to those of the OLS model (Ferrer-i-Carbonell and Frijters, 2004). Therefore, we repeat the OLS regressions and display the results in Model 2 (without health indicator) and Model 3 (with health indicator). By comparing the two models, we find that the significance level of worktime drops from 5% to 10%, while the health indicator shows a 1% significance level, implying that health abates a direct correlation between weekly working time and life satisfaction holding other factors fixed. We then calculate what proportion of life satisfaction can be explained by the influence on the health of working hours. Following the method proposed by Mo (2001), we first decompose the effect of working time on life satisfaction:

6 $$\begin{array}{l}\frac{{d\left( {{\rm {life - satisfaction}}} \right)}}{{d\left( {{\rm {worktime}}} \right)}} = \frac{{\partial \left( {{\rm {life - satisfaction}}} \right)}}{{\partial \left( {{\rm {worktime}}} \right)}} + \\ \left( {\frac{{\partial \left( {{\rm {life - satisfaction}}} \right)}}{{\partial \left( {{\rm {health}}} \right)}} \times \frac{{\partial \left( {{\rm {health}}} \right)}}{{\partial \left( {{\rm {worktime}}} \right)}}} \right)\end{array}$$

After controlling for personal characteristics and values, we regress health by worktime and find a coefficient of worktime of −0.0022 with a 5% significance level. We then calculate the impact of worktime on life satisfaction via the health change as (−0.0022) × (0.2181) = −0.0005. As the direct impact of working time on life satisfaction is (−0.0013), we get (−0.0005)/(−0.0013−0.0005) = 0.2778. This means that, on average, 27.78% of Germans’ life satisfaction can be explained by the influence of working time on their health. This result implies that health condition benefits from reduced working hours, in turn increasing life satisfaction.

Considering that the coefficients of ordered probit cannot be directly interpreted (Islam et al., 2017), we use the marginal effects measured by *δy*/*δx* to evaluate the five levels of life satisfaction. Model 4 reports the results of marginal effects, which represent the probability of changes in the dependent variables of being very unsatisfied, unsatisfied, fairly satisfied, satisfied, and very satisfied. Consistent with prior studies, we find that decreased working time generally reduces dissatisfaction and increases satisfaction. Specifically, one standard deviation (16.4218) decrease in worktime reduces the probability of very unsatisfied, unsatisfied, and fairly satisfied by 0.16% (0.0001 × 16.4218), 0.16% (0.0001 × 16.4218), and 1.15% (0.0007 × 16.4218), respectively, while also increasing the probability of satisfied and very satisfied by 0.49% (−0.0003 × 16.4218) and 0.82% (−0.0005 × 16.4218), respectively.

### Correlations of worktime and life satisfaction in three income groups

This section analyses how worktime impacts satisfaction among different income groups. We aim to deepen the analysis by detecting correlations between weekly working hours and self-reported life satisfaction for three income levels using interaction terms. The function of interaction terms is to test whether (and to what extent) the effect of one independent variable on the dependent variable depends on the magnitude of another independent variable (Ai and Norton, 2003). We, therefore, create income dummy variables—‘*Low-income*’, ‘*Mid-income*’, and ‘*High-income*’—and examine the effects of their interaction with the worktime indicator using the ordered probit model. It is important to note that interaction terms are centralised to avoid multicollinearity.

In Table 2, Models 4–6 estimate the effects of worktime on life satisfaction in the low-, mid-, and high-income scenarios. For the *Low-income* group, the coefficient is negative and significant at the 1% level. Conversely, the coefficient in the *High-income* scenario is positive at the 1% level. However, there is no evidence of a significant interaction effect in the *Mid-income* group. This result is consistent with research conducted in Germany by *Der Spiegel* during 2016–17, which found a weekly overtime working hours average of 10 h for high-income workers (with an annual salary exceeding 120,000€), compared to only about 2.1 h for low-income employees (with an annual salary below 30,000€) (Koe, 2017). This indicates that high-earners work five times the amount of overtime of low-earners, suggesting that the former prefer to work more. This may be explained by the increased income from overtime being relatively small for low-earners, whereas high-earners receive much higher rewards for extra working hours, in terms of income and/or spiritual fulfilment.

**Table 2:** Empirical analysis of the effect of worktime per week on self-reported life satisfaction at various income levels.

Variables	Dependent variable: Life satisfaction		
	Model 4: Low-income	Model 5: Mid-income	Model 6: High-income
Worktime	0.0010 (0.002)	−0.0013 (0.002)	−0.0037** (0.002)
*Three income levels*			
worktime × low-income	−0.0091*** (0.001)		
worktime × mid-income		0.0007 (0.001)	
worktime × high-income			0.0060*** (0.001)
*Individual characteristics*			
Gender	−0.0634 (0.046)	−0.0502 (0.046)	−0.0737 (0.046)
Age	0.0064*** (0.001)	0.0057*** (0.001)	0.0067*** (0.001)
Education	0.0188 (0.029)	0.0524* (0.029)	0.0135 (0.030)
Social inclusion	0.1471*** (0.025)	0.1580*** (0.025)	0.1509*** (0.025)
Health	0.3572*** (0.027)	0.3720*** (0.027)	0.3579*** (0.027)
No. of obs.	2488	2488	2488
Pseudo *R*^2^	0.0594	0.0513	0.0561

Standard errors in parentheses; *, **, and *** denote significant *p*-values at 10%, 5%, and 1% levels, respectively.

Besides, this study also tests the worktime-satisfaction nexus in different education levels and results are shown in Table S5, results showing that there is no obvious difference in worktime-satisfaction nexus for respondents with high-school, bachelor, master, or doctoral degrees.

### Effects of various levels of working hours on life satisfaction

Different levels of working hours enable people to achieve different feelings of satisfaction. For example, Americans are happier working more hours, but not Europeans (Okulicz-Kozaryn, 2011). To examine the effect in Germany, we disaggregate weekly working hours into four categories following Rudolf (2014): 1–30 h, which usually means a part-time job; 31–40 and 41–50 h, which usually implies normal full-time work; and 50+ h, which denotes working overtime. The results are displayed in Table 3. Taking all the working time categories into consideration (Model 7), the results indicate strong cross-partner effects for German husbands. A German wife’s life satisfaction is influenced by her husband’s working hours: the lower his working hours, the higher her life satisfaction. This effect deepens if the wife herself works part-time (1–30 h) (Model 8). For under-employed husbands, life satisfaction increases when their wives work more hours. This can be explained by the specialisation theory proposed by Becker (1965), whereby there is a mutually complemental effect on the market-based work and household work for the male and female in a family: ‘If the male does the lion’s share of the market work, his partner’s share of housework should be larger; conversely if he does the minority share of market work, he should do the majority share of house work’ (Akerlof and Kranton, 2000, p.188). In this situation, part-time husbands should pay more attention to family life in order to satisfy their wives. If their wives work overtime, then under-employed husbands could be happier by spending more time on housework.

**Table 3:** Effect of weekly working hours on life satisfaction in various working hour categories.

Variables	Dependent variable: Life satisfaction									
	Model 7: Hours (1–168 h)		Model 8: Hours (1–30 h)		Model 9: Hours (31–40 h)		Model 10: Hours (41–50 h)		Model 11: Hours (50 + h)	
	Male	Female	Male	Female	Male	Female	Male	Female	Male	Female
worktime	−0.0029 (0.004)	−0.0047 (0.004)	−0.0256 (0.024)	−0.0065 (0.009)	−0.0015 (0.053)	0.0013 (0.033)	0.0013 (0.024)	−0.0072 (0.037)	0.0058 (0.015)	−0.0101 (0.045)
worktime-partner	−0.0006 (0.004)	−0.0103** (0.005)	0.0343* (0.019)	−0.0238*** (0.007)	−0.0003 (0.008)	0.0014 (0.010)	−0.0018 (0.006)	−0.0012 (0.010)	−0.0056 (0.009)	0.0112 (0.021)
*Individual characteristics*										
Income	0.1136*** (0.023)	0.0737*** (0.024)	−0.0471 (0.078)	0.1028*** (0.037)	0.0746* (0.043)	0.0873* (0.046)	0.1484*** (0.036)	−0.0004 (0.054)	0.1788*** (0.064)	0.1779 (0.231)
Age	0.0024 (0.004)	0.0016 (0.004)	0.0072 (0.020)	0.0168** (0.007)	−0.0004 (0.008)	−0.0038 (0.008)	0.0045 (0.007)	−0.0039 (0.009)	0.0017 (0.010)	−0.0413 (0.029)
Education	−0.0175 (0.066)	0.0732 (0.063)	0.4060 (0.310)	0.0761 (0.112)	−0.0146 (0.119)	0.1815* (0.109)	−0.0153 (0.104)	0.0347 (0.142)	−0.1132 (0.148)	0.2221 (0.363)
Social inclusion	0.1100* (0.058)	0.1855*** (0.061)	0.2065 (0.262)	−0.0371 (0.097)	−0.0052 (0.099)	0.2468** (0.112)	0.1055 (0.098)	0.3371** (0.145)	0.2880** (0.136)	1.8807*** (0.567)
Health	0.2566*** (0.062)	0.4485*** (0.063)	0.7645*** (0.264)	0.5176*** (0.098)	0.3514*** (0.115)	0.3522*** (0.123)	0.2116** (0.098)	0.5753*** (0.153)	0.0882 (0.141)	0.3307 (0.274)
No. of obs.	518	516	37	208	162	174	232	108	87	26
Pseudo-*R*^2^	0.0588	0.0972	0.1999	0.1152	0.0464	0.0959	0.0657	0.1176	0.1016	0.4834

Standard errors in parentheses; *, **, and *** denote significant p-values at 10%, 5%, and 1% levels, respectively.

### Robustness check

This section further tests the robustness of results by changing the dependent variable and method employed. Basic regressions are presented in Table 4. Life satisfaction is replaced by happiness in Model 12 and we use the ordered logit model instead of the ordered probit model in Model 13. As can be seen, the results are very similar to the above and thus confirmed the robustness.

**Table 4:** Robustness checks.

Variables	Model 12: Happiness	Model 13: Life satisfaction
Worktime	−0.0022* (0.002)	−0.0043* (0.003)
*Individual characteristics*		
Gender	−0.0715 (0.047)	−0.1019 (0.081)
Age	0.0092*** (0.001)	0.0128*** (0.002)
Education	−0.0198 (0.030)	−0.0194 (0.051)
Social inclusion	0.1444*** (0.026)	0.2406*** (0.045)
Income	0.0590*** (0.009)	0.1257*** (0.015)
Health	0.3296*** (0.027)	0.6292*** (0.049)
No. of obs.	2489	2488
Pseudo*-R*^2^	0.0602	0.0654

Standard errors in parentheses; *, and *** denote significant *p*-values at 10% and 1% levels, respectively.

## Discussion

Multiple prior studies suggest that people are generally dissatisfied with long working hours, especially women. One explanation is that partnered women who work more hours still carry the burden of caring for the family (Schmitz and Spiess, 2021), whereas very few men mainly undertake housework such as washing and cooking. Therefore, women usually prefer a short working week (Burda et al., 2007). If society cannot provide women with enough child-care and family-care hours, as well as appropriate levels of pay, then it is unsurprising to find increasing numbers of women shortening their working hours to increase well-being.

In certain conditions, however, people who work more hours can achieve higher satisfaction. An empirical study in the US indicates that satisfaction mainly comes from work, particularly among mandatory rather than non-mandatory overtime workers, although both report higher stress than those who work no extra hours (Golden and Wiens-tuers, 2006). Rudolf (2014, p. 1156) proposes an explanation that ‘workers with these very high hours are compensated with (non-observable) non-monetary rewards, such as higher status and decision-making power (wage-employed) or higher self-determination (self-employed).’ Rothbard and Edwards (2003, p. 717) also point out an underlying explanation from the perspective of psychology: ‘instead of avoiding unpleasant role experiences, people actively try to solve the problems that make such experiences unpleasant, which requires investing time in those roles.’ This means that the problem-solving effects are triggered by unpleasant experiences (Edwards et al., 1990), and people prefer to tolerate long work hours because they anticipate increased utility in the long term.

In the US society, the distinction between life and work has long been blurred: people discuss trivialities during work, which provides a sense of belonging, while couples at home typically try to fill every minute efficiently, making this a stressful environment with too many demands (Hochschild, 1997). Several explanations of this phenomenon can be summarised here by further considering the US. First, styles of work have changed, with a gradual transfer from manual labour to mental work, which frees employees from endless toil in workshops and offices; more comfortable working environments are now preferred, such as a coffee bar for brainstorming with colleagues. This makes work more relaxed. Second, work has become more competitive, leaving employees ‘disinclined to work shorter hours because they [need] the money or [fear] losing their jobs’ (Hochschild, 1997, p. 197). Working at home becomes an unavoidable choice, which makes life busy. Finally, the market economy makes wealth an important sign of success, linked directly to life satisfaction in many circumstances (Easterlin 2001), so men and women are willing to devote more time and energy to work. Rothbard and Edwards (2003, p. 722) have already found an asymmetry relationship between life and work: ‘people are more likely to draw time from family to meet work demands than to draw time from work to meet family demands’. This implies that work impinges on life more than the reverse, and people usually anticipate a higher material reward from investing more time in work; thus, the boundary between work and life becomes blurred.

Overall satisfaction is affected by both positive and negative effects. On the one hand, extra monetary compensation for workers may enhance well-being, providing a sense of achievement; on the other hand, working more hours may interfere with family life, increasing mental stress and even harming health. The superposition effect of the two influences decides the final result. In fact, relative preference for work and leisure varies across multiple countries and regions due to different economic, social, and cultural contexts. For example, Europeans and Americans are two typical contrasting cases that attract numerous researchers’ attention. Evidence shows that the former like to enjoy their leisure time with family and friends, sports, watching TV, or developing their own interests at home; by contrast, the latter prefer to spend more time working to develop their careers (Landsburg, 2006). Besides the roles in Europe of marginal tax rates (Prescott, 2004) and powerful labour unions (Alesina et al., 2006), another possible explanation is that Europeans place more value on the process of work, whereas Americans prioritise work outcomes. As Okulicz-Kozaryn (2011, p. 225) argues, ‘Europeans work to live and Americans live to work’. Furthermore, personal characteristics are also influential. People living in poor communities may have a lower willingness to work long hours compared to those living in rich communities; Men and women also have different attitudes on the distribution of time between work and life (Rothbard and Edwards, 2003; Clark and Senik, 2006; Rudolf, 2014).

In general, people’s overall satisfaction depends on how they balance their work and life, in terms of not only their distribution of time but also their ability to escape work pressure by ‘buying’ life satisfaction (Whillans et al., 2017). As Easterlin (2004, p. 33) argues, ‘Most people could increase their happiness by devoting less time to making money, and more time to nonpecuniary goals such as family life and health’.

## Conclusions and policy implications

### Conclusions

This study investigated the effect of reduced working hours on life satisfaction for Germans using an ordered probit model, based on the ESS data. The results show that working time is negatively associated with life satisfaction, which implies that people generally prefer a short working week. Health is confirmed to be the mediating variable between working time and life satisfaction: for the average German, about 28% of life satisfaction can be explained by the effect of working time on their health. In terms of income levels, high-earners tend to work longer while low-earners typically work fewer hours. However, middle-earners show no clear preference for working hours. Cross-partner effects are also confirmed: the shorter working week of German husbands can enhance the wives’ life satisfaction, whereas a longer working week for wives can improve their husbands’ life satisfaction. Robustness checks of replacing the dependent variable of life satisfaction with happiness and using the ordered logit model instead of the ordered probit model confirm the validity of our results. These findings complement conventional views on working time and life satisfaction.

### Policy implications

Based on the study’s findings, several policies can be proposed. On one side, regulations limiting hours of work and protecting employees’ health should be further enacted. Since health is an important factor in the process of working hours influencing life satisfaction, good physical and mental health can significantly improve life satisfaction. Working too long or too little could have a detrimental effect on satisfaction; it is important to confine working hours to moderate levels in order to satisfy workers. On the other side, gender differences should be taken into account in policy-making. This study reinforces prior findings that men and women have different attitudes toward their preferred working hours. Generally speaking, women tend to work part-time to combine work with caring for their families, while men are inclined to work full-time or even longer to pursue success. Against this backdrop, different working time regulations for men and women should be considered. Except for the difference between maternity leave for women and paternity leave for men, the flexible working time regime is another good case in point. For women in the job market, absenteeism would be significantly lower with a flexible, rather than fixed, work schedule (Krausz and Freibach, 1983), and flexible working can play the same role as part-time work to reduce pressure and increase well-being (Russell et al., 2009). Indeed, for both male and female employees, flexible workers usually report higher levels of well-being than their non-flexible counterparts (Costa et al., 2004; Kelliher and Anderson, 2009; Schmitz and Spiess, 2021).

Three future research directions are highlighted here. First, more sophisticated techniques can be employed to avoid the potential endogeneity problem generated by reverse causalities, such as the fixed-effect model (Rudolf, 2014). Second, a cross-sectional or panel threshold model can be used to find out the thresholds beyond which longer or shorter working hours may decrease life satisfaction. Third, besides health, other factors potentially play essential roles in the process through which working time affects life satisfaction. For example, social trust (Awaworyi Churchill and Mishra, 2017; Lu et al., 2020), feeling of safety (Kuroki, 2013; Staubli et al., 2014), and even digitalisation (Bolli and Pusterla, 2022; Elmassah and Hassanein, 2022) are potential driving forces of life satisfaction. They are all interesting themes that deserve further research.

## Supplementary information

Supplementary Information

Data do file

Data dta file

## Data Availability

The datasets generated during and/or analysed during the current study are available from the corresponding author on reasonable request.
