# Supplementary material for: Exploring the promoting effect of working time reduction on life satisfaction using Germany as a case study
Source: Humanit Soc Sci Commun. 2022 Dec 17;9(1):454. doi: 10.1057/s41599-022-01480-2 (PMC9759043; doi:10.1057/s41599-022-01480-2)
Supplement: Supplementary file 1 — Supplementary Information [file 41599_2022_1480_MOESM1_ESM.docx]

**Exploring the promoting effect of working time reduction on life satisfaction using Germany as a case study**

Qinglong Shao

Institute of Chinese Studies, Freie Universität Berlin, Fabeckstr. 23-25, 14195 Berlin, Germany

Correspondence: qinglong.shao@fu-berlin.de

Table S1 presents the survey questions and descriptions of the variables from the German European Social Survey (ESS) dataset. The raw life satisfaction data of Germany are presented in Table S2. The numbers and overall life satisfaction scores appear to be skewed towards the top of the possible answer distribution. In other words, individuals seem to answer optimistically. Almost half of respondents say they are satisfied and a quarter are very satisfied. Since the large proportion of unmarried partners, the unmarried report similar satisfaction to the legally married, showing that marriage is no longer a determinant of life satisfaction. The same applies to the sex categories. Whatever the appropriate interpretation of this pattern, it is clear that part-time employees (work less than 30 hours per week) and low-earners are much less content in Germany. These are two of the most negative life scenarios. By contrast, the fulfilment of long working hours and high income are two of the factors most strongly associated with high life satisfaction.

Table S3 provides descriptive statistics of the variables of interest in our estimation sample. Table S3 demonstrates considerable variations throughout the observations for the dependent, independent, and control variables. Pairwise correlations for the dependent variables and all the explanatory variables are reported in Table S4**.** The results illustrate two facts: first, most variables are significantly correlated at the 10 percent level; second, working time is negatively correlated with life satisfaction, as well as other explanatory variables except for gender and age. The observations highlight the importance of careful multivariate econometric analysis.

| **Table S1 Survey questions and descriptions of the variables from the German ESS dataset.** | | |
| --- | --- | --- |
| **Variables** | **Survey questions** | **Responses** |
| *Life satisfaction* | B27: All things considered, how satisfied are you with your life as a whole nowadays? | ‘Very unsatisfied’ = 1; ‘unsatisfied’ = 2; ‘fairly satisfied’ = 3; ‘satisfied’ = 4; ‘very satisfied’ = 5 |
| *Happiness* | C1: Taking all things together, how happy would you say you are? | ‘Very unhappy’ = 1; ‘unhappy’ = 2; ‘fairly happy’ = 3; ‘happy’ = 4; ‘very happy’ = 5 |
| *Worktime* | F30: Regardless of your basic or contracted hours, how many hours do/did you normally work a week (in your main job), including any paid or unpaid overtime? | Hours worked per week, between 0 and 168 hours |
| *Income* | F41: Which letter describes your household’s total income, after tax and compulsory deductions, from all sources? | Scored from 0 to 10, where 0 means ‘extremely low’ and 10 means ‘extremely high’, for weekly, monthly, and annual amounts |
| *Health* | C7: How is your health in general? | ‘Very bad’ = 1; ‘bad’ = 2; ‘fair’ = 3; ‘good’ = 4; ‘very good’ = 5 |
| *Gender* | F2: Sex | ‘Male’ = 1; ‘Female’ = 0 |
| *Age* | F3: Age | Calculated by birth year |
| *Education* | F15: What is the highest level of education you have successfully completed? | ‘High school or lower’ = 1; ‘bachelor degree’ = 2; ‘master degree’ = 3; ‘doctoral degree’ = 4 |
| *Social inclusion* | C4: Compared to other people of your age, how often would you say you take part in social activities? | ‘Much less than most’ = 1; ‘Less than most’ = 2; ‘About the same’ = 3; ‘More than most’ = 4; ‘Much more than most’ = 5 |
| **Note**: In the questionnaire *life satisfaction* is scored from 0 to 10, where 0 means ‘extremely dissatisfied’ and 10 means ‘extremely satisfied’. To better illustrate the marginal effects, we have recoded the scale so that a higher value indicates a higher level of life satisfaction: from the original responses, 0 is defined as ‘very unsatisfied’; 1 and 2 are ‘unsatisfied’; 3, 4, 5, 6, and 7 are ‘fairly satisfied’; 8 and 9 are ‘satisfied’; and 10 is ‘very satisfied’.  **Source**: ESS (2016b). | | |

| **Table S2** **Life satisfaction distribution in terms of marital status, sex, income and working time categories.** | | | | | | | | | | | | |
| --- | --- | --- | --- | --- | --- | --- | --- | --- | --- | --- | --- | --- |
| **Life satisfaction** | **All**  *(%)* | **Marital Status** | | **Sex** | | **Income levels** | | | **Working time categories** | | | |
|  |  | *Married*  *(%)* | *Un-married*  *(%)* | *Male*  *(%)* | *Female*  *(%)* | *Low*  *(%)* | *Middle*  *(%)* | *High*  *(%)* | *1-30 h (%)* | *31-40 h (%)* | *41-50 h (%)* | *50+ h (%)* |
| Very unsatisfied | 0.77 | 5 | 0.68 | 0.53 | 1.04 | 1.80 | 0.26 | 0.52 | 1.34 | 0.65 | 0.49 | 0.65 |
| Unsatisfied | 1.90 | 5 | 1.83 | 2.39 | 1.34 | 5.39 | 1.31 | 0.68 | 1.74 | 2.18 | 1.72 | 1.92 |
| Fair satisfied | 35.24 | 46.67 | 34.99 | 34.62 | 35.95 | 49.02 | 39.03 | 26.45 | 32.93 | 36.71 | 35.26 | 37.82 |
| Satisfied | 48.07 | 28.33 | 48.55 | 49.50 | 46.46 | 32.35 | 44.81 | 58.53 | 47.79 | 47.06 | 49.87 | 45.83 |
| Very satisfied | 14.02 | 0.15 | 13.95 | 12.96 | 15.21 | 11.44 | 14.59 | 13.82 | 16.20 | 13.40 | 12.65 | 13.78 |
| **Note**: Based on 2,852 observations. | | | | | | | | | | | | |

| **Table S3** **Descriptive statistics.** | | | | | | | |
| --- | --- | --- | --- | --- | --- | --- | --- |
| **Variables** | **Mean** | **S.D.** | **Min** | **P25** | **P50** | **P75** | **Max** |
| *Life satisfaction* | 3.7266 | 0.7507 | 1 | 3 | 4 | 4 | 5 |
| *Worktime* | 36.7222 | 16.4218 | 0 | 30 | 40 | 45 | 100 |
| *Income* | 5.8614 | 2.7925 | 1 | 4 | 6 | 8 | 10 |
| *Health* | 3.6501 | 0.9018 | 1 | 3 | 4 | 4 | 5 |
| *Gender* | 0.52875 | 0.4993 | 0 | 0 | 1 | 1 | 1 |
| *Age* | 48.5577 | 18.4977 | 15 | 33 | 50 | 63 | 94 |
| *Education* | 1.4263 | 0.7821 | 1 | 1 | 1 | 2 | 4 |
| *Social inclusion* | 2.72439 | 0.9048 | 1 | 2 | 3 | 3 | 5 |
| **Note**: S.D. is the standard deviation; P25, P50, and P75 respectively denote the 25% percentile, median, and 75% percentile. | | | | | | | |

| **Table S4** **Correlation matrix.** | | | | | | | | | |
| --- | --- | --- | --- | --- | --- | --- | --- | --- | --- |
| **Variables** |  | **1** | **2** | **3** | **4** | **5** | **6** | **7** | **8** |
| *Life satisfaction* | **1** | 1.0000 |  |  |  |  |  |  |  |
| *Worktime* | **2** | −0.0267* | 1.0000 |  |  |  |  |  |  |
| *Income* | **3** | 0.2145* | 0.0905* | 1.0000 |  |  |  |  |  |
| *Health* | **4** | 0.2866* | −0.0897* | 0.2171* | 1.0000 |  |  |  |  |
| *Gender* | **5** | −0.0099 | 0.2343* | 0.0627* | 0.0424* | 1.0000 |  |  |  |
| *Age* | **6** | 0.0076 | 0.2874* | −0.1177* | −0.2654* | −0.0071 | 1.0000 |  |  |
| *Education* | **7** | 0.0686* | 0.1242* | 0.2689* | 0.0701* | 0.0126 | 0.0851* | 1.0000 |  |
| *Social inclusion* | **8** | 0.1788* | −0.0324* | 0.1500* | 0.1597* | 0.0374* | 0.0018 | 0.0485* | 1.0000 |
| **Note**: * denote correlation is significant at the 0.10 level (2-tailed). | | | | | | | | | |

**Correlations of worktime and life satisfaction in four education levels.** As can be seen from Table S5, the four interaction terms show no significant signs, manifesting that there is no obvious difference in worktime-satisfaction nexus for respondents with high-school, bachelor, master, or doctoral degrees.

| **Table S5 Empirical analysis of the effect of worktime per week on self-reported life satisfaction at various education levels.** | | | | |
| --- | --- | --- | --- | --- |
| **Variables** | **Dependent Variable: *Life Satisfaction*** | | | |
|  | **Model 7:**  **High school or lower** | **Model 8:**  **Bachelor degree** | **Model 9:**  **Master degree** | **Model 10:**  **Doctoral degree** |
| *Worktime* | −0.0031*  (0.002) | −0.0021  (0.002) | −0.0022  (0.002) | −0.0023  (0.002) |
| **Four education levels** | | | | |
| *worktime×*  *high school or lower* | 0.0011  (0.001) |  |  |  |
| *worktime×*  *bachelor degree* |  | −0.0019  (0.002) |  |  |
| *worktime×*  *master degree* |  |  | −0.0003  (0.002) |  |
| *worktime×*  *doctoral degree* |  |  |  | 0.0006  (0.004) |
| **Individual characteristics** | | | | |
| *Gender* | −0.0614  (0.046) | −0.0584  (0.046) | −0.0622  (0.046) | −0.0617  (0.046) |
| *Age* | 0.0073***  (0.001) | 0.0072***  (0.001) | 0.0072***  (0.001) | 0.0072***  (0.001) |
| *Income* | 0.0698***  (0.009) | 0.0687***  (0.008) | 0.0681***  (0.009) | 0.0676***  (0.008) |
| *Social inclusion* | 0.1365***  (0.025) | 0.1366***  (0.025) | 0.1364***  (0.025) | 0.1364***  (0.025) |
| *Health* | 0.3456***  (0.027) | 0.3450***  (0.027) | 0.3446***  (0.027) | 0.3443***  (0.027) |
| *No. of Obs.* | 2,488 | 2,488 | 2,488 | 2,488 |
| *Pseudo R^2^* | 0.0627 | 0.0628 | 0.0626 | 0.0626 |
| **Note**: Standard errors in parentheses; *, **, and *** denote significant p-values at 10%, 5%, and 1% levels, respectively. | | | | |

**Reference:**

ESS (2017) ESS Round 8 Source Questionnaire. 94
